# MicroRNA and Cardiovascular Disease

**DOI:** 10.1155/2015/734380

**Published:** 2015-07-21

**Authors:** Xiao-Bo Liao, Vinicio A. de Jesus Perez, Magdalena Król, Chi-Hsiao Yeh, Ling-Qing Yuan

**Affiliations:** ^1^Departments of Cardiothoracic Surgery, Central South University, Changsha 410011, China; ^2^Division of Pulmonary and Critical Care Medicine, Stanford University Medical Center, Stanford, CA 94305, USA; ^3^Warsaw University of Life Sciences, 02-787 Warsaw, Poland; ^4^Chang Gung Memorial Hospital, Keelung 20445, Taiwan; ^5^Institutes of Metabolism and Endocrinology, Central South University, Changsha 410011, China

Cardiovascular disease (CVD) is the leading cause of death and a major financial burden in most developed countries around the world, resulting in an estimated death toll of 17 million people in 2011. During the past decade, there have been significant advances in our knowledge of the pathogenesis, diagnosis, and treatment in CVD. Recently, microRNAs (miRNAs) have emerged as one of the most important regulators of gene function and tissue homeostasis that appear to be amenable for development of novel therapies to treat deleterious mutations and restore cellular function in various diseases. Despite the major discoveries made in the past decade, there are still major gaps in our knowledge as to how miRNAs are involved in preserving normal cardiovascular function and how dysregulation in their expression or biological function results in CVD. Available studies strongly support that miRNAs could be targeted in various ways to restore normal cardiovascular function and could find a role in the clinic as therapeutic tools to improve our ability to prevent and effectively treat CVD, likely resulting in improved outcomes and reduced rates of mortality from these diseases. Our current special issue presents a series of original researches and reviews on recent advances in miRNAs regulating the pathophysiology aspects of CVD that will hopefully set the stage for future research initiatives aimed at expanding our understanding of these important mediators of cardiovascular function.

Among the studies included in this special issue, some researchers investigate the miRNA function in different CVD. M. Torrado et al. used the right atrial tachypacing to establish an atrial fibrillation pig model and show that the abnormal expression of miRNAs is associated with modulation of transcription factors in left atrium of this animal model. Their study provides critical insight into how the microRNA-transcription factor regulation network underlies the onset of paroxysmal atrial fibrillation. In another article, A. M. dos Santos et al. report that laminar shear stress (LSS) has a protective effect on human umbilical vein endothelial cells (HUVECs) via concomitant increased expression of miR-126, vascular cell adhesion molecule-1 (VCAM-1), and syndecan-4 (SDC-4). This is supported by in vivo studies using Apo-E KO/CKD mice, a well-established animal model of atherosclerosis and aortic calcification. We see that gain- and loss-of-function studies of miR-126 result in significant changes in the expression of several cytokines by HUVEC that suggest flow might have anti-inflammatory and antiatherosclerosis effect on the endothelium of the systemic circulation. The study by Z.-Y. Xia et al. reveals an interesting interaction between miR-3960/miR-2861 and Runx2 in vascular smooth muscle cells, which regulates osteogenic differentiation of VSMCs. Their studies identify a novel mechanism by which miR-3960/miR-2861 target histone deacetylase 5 or Homeobox A2 that could modify the expression of Runx2 and create a feedback mechanism targeting miR-3960/miR-2861 itself. The authors speculate that this complex regulation feedback loop might play a pivotal role in osteogenic differentiation of VSMCs and contribute to vascular calcification in CVD.

A major focus of studies into miRNA function is the identification of the genes targeted in target cells and tissues. E. Lozano-Velasco et al. report that miRNA might have distinct effects on the same target gene in different cells as shown when miR-125 is overexpressed in HL1 atrial cardiomyocytes where it increases Mef2d while suppressing this gene in Sol8 cells. Two or more miRNAs could constitute a “microRNA cluster,” which can have important roles in controlling physiological and pathological conditions. X. Zhang et al. found miR-17-92, miR-106a-363, and miR-106b-25 clusters were differently expressed between aged and young adult mouse heart and glucose stress could influence their upregulation or downregulation. Using computational algorithms for miRNAs, the authors postulate miR-17-92 cluster and its paralogs might regulate Cdc42-SRF pathway protein components involved in cardiac structure and function. This paper shows that miRNA cluster plays an important role in cardiac morphology and response to environmental stimuli.

There are still several interesting reviews about recent progress on miRNAs and CVD. M. Notari et al. give an excellent review, which discusses the relationship between miRNAs and cardiac development, myocardial regeneration, and cardiovascular disease. They also discuss the potential use of miRNA as a therapeutic option for CVD and the obstacles that must be overcome before these agents reach the clinic. S.-S. Wu et al.'s paper reviews the role of epigenetics in the pathogenesis of arterial calcification. They discuss the relationship between DNA methylation, histone modifications, and microRNAs in regulation of arterial calcification. miR-221 and miR-222 are transcribed from the same miRNA cluster, exhibit high sequence similarity, and share similar target genes. Through discussing the role of miR-221/miR-222 in endothelial cells and VSMCs, D. A. Chistiakov et al. review their role in physiological and atherosclerotic vascular remodeling. W. Zhao et al. discuss the effect of miR-143/145 on VSMCs, endothelial cells, and plasma and suggest that miR-143/145 might be a potential drug target for CVD. X. Fu et al. review some miRNAs involved in the pathogenesis of aortic aneurysm and suggest several miRNAs that might be used as potential diagnostic and prognostic biomarker as well as therapeutic targets for aortic aneurysms. R. Shi et al. review recent studies that stress an essential role of miR-223 as both a regulator and biomarker for platelet reactivity and major cardiovascular events. They suggested miR-223 might be a potential diagnostic tool for recognizing high on-treatment platelet reactivity in clinical practice.

